# Pivotal vascular homeostatic role for endothelium-derived C-type natriuretic peptide (CNP)

**DOI:** 10.1186/2050-6511-16-S1-A27

**Published:** 2015-09-02

**Authors:** Adrian J Hobbs

**Affiliations:** 1 William Harvey Research Institute, London, UK

## Background

The endothelium plays a fundamental role in maintaining vascular homeostasis by releasing factors that regulate local blood flow, systemic blood pressure, and the reactivity of leukocytes and platelets. Accordingly, endothelial dysfunction underpins many cardiovascular diseases, including hypertension, myocardial infarction and stroke. C-type natriuretic peptide (CNP) is a paracrine mediator that possesses a unique vaso- and cardio- protective pharmacodynamic profile; however, there is a paucity of information regarding a physiological role for CNP within the cardiovascular system.

## Methods and principal results

Herein, we generated a novel endothelial cell-specific CNP knockout (ecCNP KO) mouse to investigate a potential vasoprotective role of endogenous CNP in vivo. Mice lacking endothelial CNP exhibited a fundamental, multi-faceted vascular dysfunction, including impaired endothelium-dependent dilatation, hypertension, increased leukocyte & platelet reactivity, accelerated atherogenesis and aneurysm. Moreover, the aberrant vascular phenotype observed in ecCNP KO animals was more closely recapitulated in mice with global deletion of natriuretic peptide receptor (NPR)-C than animals lacking guanylate cyclase-coupled NPR-B. In accord, we designed & developed a series of novel, small molecule NPR-C agonists, which are vasorelaxant in vitro and reduce blood pressure in vivo.

## Conclusion

These data identify a mechanism explaining genome-wide association studies linking the NPR-C (Npr3) and Furin (a pro-protein convertase obligatory in the bioactivation of CNP) loci with hypertension, and establish the importance of CNP-NPR-C signalling in preserving vascular homoeostasis and as a disease-modifying drug target.

This work was supported by the Wellcome Trust and UCL Business PLC.

